# Novel t(1;2)(p36.1;q23) and t(7;19)(q32;q13.3) chromosomal translocations in ischemic fasciitis: expanding the spectrum of pseudosarcomatous lesions with clonal pathogenetic link

**DOI:** 10.1186/s13000-018-0695-y

**Published:** 2018-03-02

**Authors:** Taha Sachak, Nyla A. Heerema, Joel Mayerson, Jason E. Payne, Anil Parwani, O. Hans Iwenofu

**Affiliations:** 1 Department of Pathology and Laboratory Medicine, Wexner Medical Center at The Ohio State University, 410 West 10th Avenue, Columbus, OH 43210 USA; 2 Department of Orthopaedics, Wexner Medical Center at The Ohio State University, Columbus, OH USA; 3 Department of Radiology, Wexner Medical Center at The Ohio State University, Columbus, OH USA

**Keywords:** Ischemic fasciitis, Pseudosarcoma, Clonal chromosomal translocations

## Abstract

**Background:**

Ischemic fasciitis is a distinctive pseudosarcomatous entity with a marked predilection for elderly and physically debilitated or immobilized patients. The etiology of these lesions is unknown but felt to be related to ischemic vascular events.

**Case presentation:**

Herein, we report for the first time, two cytogenetic translocations, t(1;2)(p36.1;q23) and t(7;19)(q32;q13.3) in a 75 year-old ambulating female with a history of left total hip arthroplasty 20 years ago.

**Conclusion:**

These translocations suggest a possible clonal pathogenetic link though their significance remains to be established.

## Background

Ischemic fasciitis, also known as atypical decubital fibroplasia, is a pseudosarcomatous entity that is known to typically occur in elderly patients and patients who have had a long standing history of debility [1, 2]. However, cases of non-debilitated patients have been reported in the past [3], as it is the case with our patient. The incidence of ischemic fasciitis peaks in the eighth and ninth decades but there is a wide age distribution with the disease being reported as young as 23 years, and there is a slight male predominance [4]. They predominantly involve the soft tissue and consist of a fibroblastic and myofibroblastic proliferation in a poorly circumscribed fashion and infiltrating growth pattern thereby simulating a sarcomatous growth clinically, radiologically and histopathologically [5]. To the best of our knowledge there are no reported cytogenetic abnormalities associated with this entity and thus, for the first time, we report two distinct, but novel cytogenetic translocations, t(1;2) (p36.1;q23) and t(7;19) (q32;q13.3) in a 75 year-old ambulating female with a history of left total hip arthroplasty 20 years ago.

## Case presentation

**Fig. 1:**
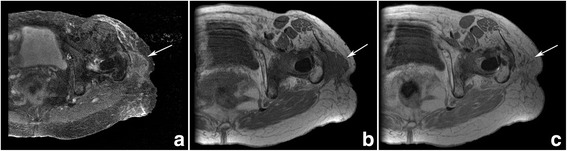
(**a)** Axial MR STIR images of the left hip at the level of the greater trochanter show an irregular subcutaneous mass (white arrow) involving the iliotibial band and abutting the greater trochanter. The mass is primarily hyperintense on fluid-sensitive sequences. On axial T1-weighted pre-gadolinium (**b)** the mass is isointense to muscle and enhances avidly following contrast administration on post-gadolinium images (**c**)

A 75-year-old female was referred from an outside institution for evaluation of a suspected soft tissue tumor. She first noticed a painless soft tissue mass on her left hip approximately 6 weeks prior to presentation. She denied having pain in the identified area without a change in size and firmness of the mass. Her past surgical history is significant for a left total hip arthroplasty 20 years ago, which hadn’t required any revision surgeries. She denied any pain with ambulation, activities of daily living, and work. Magnetic resonance imaging (MRI) of the left hip showed an ovoid signal abnormality within the subcutaneous fat with a heterogenous enhancement of this structure (Fig. 1a-c). A fine needle aspiration biopsy was performed and results were suspicious for sarcomatous proliferation. The patient subsequently underwent a surgical resection of this mass. The patient is now 2.5 years post-surgical resection with no evidence of recurrent or metastatic disease.

**Fig. 2:**
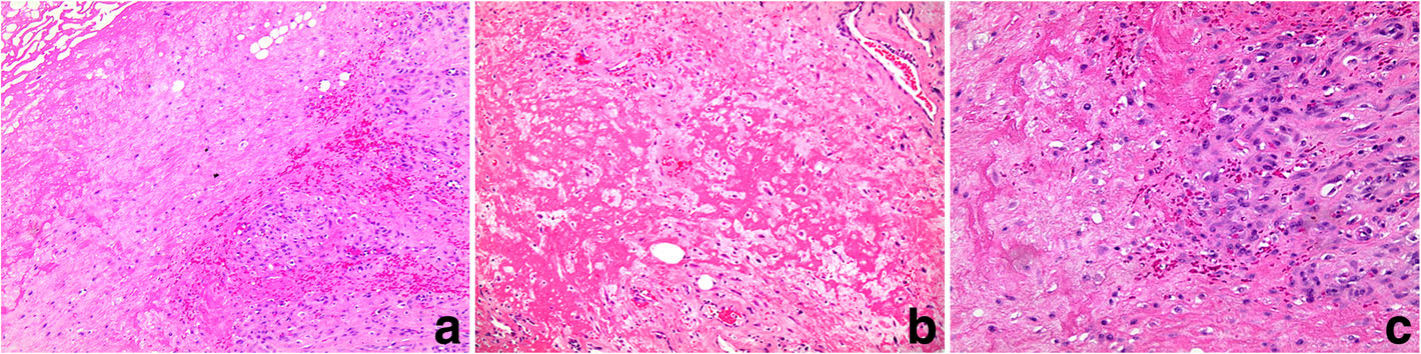
**a** A low power image of Hematoxylin and Eosin (H&E) stained section is depicted here, demonstrating pattern of zonation with fibrinoid matrix deposition. Also, extravasation of red blood cells, proliferating atypical fibroblasts and so-called “ghosted” fat cells are seen. **b** A high power view of zonal fibrinoid matrix deposition with intervening proliferative fibroblasts is shown; a non-thrombosed vessel is present (top right). **c** A higher power view demonstrating plump and proliferative fibroblasts adjacent to focal fibrinoid changes

Grossly, the excision specimen consisted of a 5.8 × 2.7 × 2.4 cm densely fibrotic tan-white poorly – circumscribed mass. The cut surfaces showed an orange and red slightly marbled surface with hemorrhage accounting for approximately 10 %. Noteworthy, were finger-like fibrotic extensions to the deep margin where the mass possibly involved the fascial plane. Microscopically, the findings were considered prototypic for ischemic fasciitis, consisting of a distinct zonal pattern observed at low power with fibrinoid matrix deposition surrounded by an area composed of reactive/atypical appearing fibroblasts and granulation tissue. Some of the atypical fibroblastic/myofibroblastic cells had a ganglion cell-like appearance akin to those described in proliferative fasciitis. There were also focal areas showing myxoid alteration, giant cell reaction, and extravasation of red blood cells (Fig. 2a-c).

A representative portion of tumor was minced and treated with collagenase (Sigma Aldrich, Inc) for 4 h and cultured on cover-slips for 7–9 days for cytogenetic analysis. Harvest consisted of colcemid (0.08 mg/ml) for 3 h. The coverslips were fixed in-situ*.* Hypotonic was 0.075 M KCL (Sigma Aldrich, Inc) for 20 min at 37 °C, and fixation was with 3:1 methanol:acetic acid. The cells were G-banded using trypsin (Gibco Invitrogen), and stained with Wright stain (Sigma Aldrich, Inc) according to standard laboratory procedures. All metaphases were completely analyzed. Karyotypes were described according to the ISCN 2013 standard [6].

**Fig. 3:**
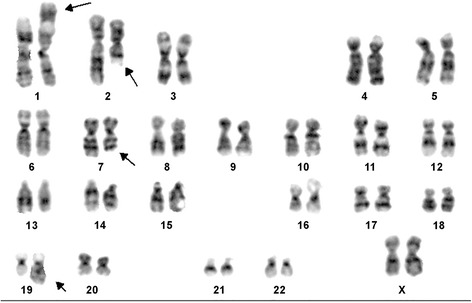
G-Banded Karyotype showing 46,XX,t(1;2)(p36.1;q23),t(7;19)(q32;q13.3). Arrows indicate breakpoints

The cytogenetic analysis of the sample showed an abnormal karyotype with a translocation between chromosome 1 and 2, and a translocation between chromosome 7 and 19. A total of 20 cells were karyotyped and 5 cells (25%) contained cytogenetic abnormalities. A full karyotype present was as follows: 46,XX,t(1;2)(p36.1;q23) [3]/46,idem,t(7;19)(q32;q13.3)[2]/46,XX[12]/nonclonal[3] (Fig. 3).

## Discussion and conclusion

Ischemic fasciitis is a pseudosarcomatous proliferation that shows predilection to older or debilitated patients [1]. The term is synonymous with atypical decubital fibroplasia as described by Montgomery et al. in their 1992 paper [1] and clinically presents as a painless mass that typically occurs over bony protuberances [7] such as sacral, greater trochanter and limb girdles [8]. Grossly, it is poorly circumscribed, infiltrative, and can be multinodular. The subcutaneous tissue is predominantly involved; however, dermal involvement has been described. Histologically, there is a classic zonal distribution of coagulative type necrosis surrounded by thin ectatic vessels and proliferating fibroblasts. The pathogenesis of ischemic fasciitis is not fully understood, but it is believed to arise from local ischemia to an area due to prolonged vascular compromise [2].

To our knowledge this is the first case of cytogenetic aberrations reported in a case of ischemic fasciitis. In our case, the cytogenetic analysis was done on a previously untreated ischemic fasciitis and demonstrated a novel chromosomal translocation between chromosomes 1 and 2, and a translocation between chromosome 7 and 19. Similar to other pseudosarcomatous lesions, entities such as nodular fasciitis, proliferative fasciitis and myositis have long been held to represent reactive processes rather than neoplastic. However, the discovery of *MYH9-USP6* fusion gene as recurrent event in nodular fasciitis; trisomy 2 and t(6;14)(q23;q32) in proliferative fasciitis and myositis respectively suggests a putative clonal neoplastic origin rather than reactive process [9–11]. Furthermore, aneurysmal bone cyst (ABC), a benign but recurrent locally aggressive bone lesion, has been found to harbor a recurring chromosomal translocation involving t(16;17)(q22;p13) [12] .This translocation results in the fusion of osteoblast cadherin 11 gene (*CDH11*) promoter region on 16q22 in juxtaposition with ubiquitin-specific protease (*USP6*) on 17p13, implicating a novel oncogenic driver in the pathogenesis of ABC [12]. Importantly, USP6 and CDH11 gene rearrangements or their variants thereof are present only in the spindle cells of primary ABC but not in the secondary ABC, suggesting that this fusion gene (and variants) is specific for this entity and pathogenetically relevant only in the context of primary aneurysmal bone cyst [13].

In our case, 25% of the cells had cytogenetic abnormality and all the cells with clonal chromosomal aberration were found on the coverslips, suggesting that this is intrinsic to the tumor and thus the possibility this is a cultural artifact is very unlikely. Secondly, the cytogenetic abnormalities were present in different coverslips. Importantly, all 5 abnormal cells had t(1;2) while 2 cells had t(7;19). Interestingly, we note the existence of two unrelated chromosomal abnormalities within the same tumor. This is a rather uncommon phenomenon but we believe that this may represent acquired changes secondary to ischemic damage to the fibroblasts. Similarly, there is an on-going debate that the trisomy 2 in proliferative fasciitis is only an age-related change rather than a bona-fide clonal event in proliferative fasciitis. Whether this is a similar phenomenon remains to be established. Previously, anomalies involving chromosome 7 and 19 although with different breakpoints; t(7;19)(q22;q13) have been described in a case of pseudomyogenic hemangioendothelioma (PHE) [14]. PHE is an extremely rare soft tissue tumor that frequently arises in young adult males. Only recently, a balanced translocation, t(7;19)(q22;q13), resulting in a fusion of the *SERPINE1* and *FOSB* genes has been demonstrated as the sole molecular genetic abnormality in PHE [15].

At present, the knowledge about the significance and implications of these cytogenetic aberrations associated with ischemic fasciitis are not known. This is the first case to the best of our knowledge that describes these unique translocations. This potentially raises a very important question as to whether this is fundamentally a clonal neoplastic process or a reactive ischemic process characterized by vascular compromise. The relative rarity of this lesion itself, coupled with the occurrence of this cytogenetic abnormality makes it difficult to ascertain the significance of these findings.

In conclusion, we present for the first time, a novel case of clonal chromosomal translocations between chromosomes 1 and 2, and translocation between chromosomes 7 and 19; t(1;2)(p36.1;q23) and t(7;19)(q32;q13.3), respectively occurring in ischemic fasciitis of the lower extremity. The significance of these clonal cytogenetic translocations remains to be unravelled.

## Abbreviations

ISCN: International System for Human Cytogenetic Nomenclature
MRI: Magnetic Resonance Imaging
PHE: Pseudomyogenic Hemangioendothelioma

## References

[1] Montgomery EA, Meis JM, Mitchell MS (1992). A typical decubital fibroplasia. A distinctive fibroblastic pseudotumor occurring in debilitated patients. Am J Surg Pathol.

[2] Baranzelli MC, Lecomte-Houcke M, De Saint Maur P (1996). Atypical decubital fibroplasia: a recent entity. A propos of a case of an adolescent girl. Bull Cancer.

[3] Wader J, Gajbi N, Kumbhar S (2013). Ischaemic fasciitis: a very rare entity with unusual presentation. J Clin Diagn Res.

[4] Liegl B, Fletcher CDM (2008). Ischemic fasciitis: analysis of 44 cases indicating an inconsistent association with immobility or debilitation. Am J Surg Pathol.

[5] Kendall BS, Liang CY, Lancaster KJ (1997). Ischemic fasciitis. Report of a case with fine needle aspiration findings. Acta Cytol.

[6] Simons A, Shaffer LG, Hastings RJ (2013). Cytogenetic nomenclature: changes in the ISCN 2013 compared to the 2009 edition. Cytogenet Genome Res.

[7] Oguejiofo LN, Sheyner I, Stover KT (2011). Ischemic fasciitis in an 85-year-old debilitated female. J Am Med Dir Assoc.

[8] Liegl-Atzwanger B, CDM F, Bridge JA, Hogendorn PCW, Mertens F (2013). Ischemic fasciits. WHO Classification of Tumours of Soft Tissue and Bone.

[9] Erikson-Johnson MR, Chou MM, Evers BR (2011). Nodular fasciitis: a novel model of transient neoplasia induced by MYH9-USP6 gene fusion. Lab Investig.

[10] Dembinski A, Bridge JA, Neff JR, Berger C, Sanberg AA (1992). Trisomy 2 in proliferative fasciitis. Cancer Genet Cytogenet.

[11] McComb EN, Neff JR, Johansson SL, Nelson M, Bridge JA (1997). Chromosomal anomalies in a case of proliferative myositis. Cancer Genet Cytogenet.

[12] Oliveira AM, HIS BL, Weremowicz S (2004). USP6 (Tre2) fusion oncogenes in aneurysmal bone cyst. Cancer Res.

[13] Oliveira AM, Perez-Atayde AR, Inwards CY (2004). USP6 and CDH11 oncogenes identify the neoplasticcell in primary aneurysmal bone cysts and are absent in so-called secondary aneurysmal bone cysts. Am J Pathol.

[14] Trombetta D, Magnusson L, von Steyern FV (2011). Translocation t(7;19)(q22;q13)−a recurrent chromosome aberration in pseudomyogenic hemangioendothelioma?. Cancer Genet.

[15] Walther C, Tayebwa J, Lilljebjörn H (2014). A novel *SERPINE1-FOSB* fusion gene results in transcriptional up-regulation of FOSB in pseudomyogenic haemangioendothelioma. J Pathol.

